# Clinical Presentation, Treatment Outcomes, and Resistance-related Factors in South American Women with Low-risk Postmolar Gestational Trophoblastic Neoplasia

**DOI:** 10.1055/s-0042-1748974

**Published:** 2022-06-27

**Authors:** Luz Angela Correa Ramírez, Izildinha Maestá, María Inés Bianconi, Gustavo Jankilevich, Silvina Otero, Carlos Raúl Villegas Mejía, Rafael Cortés-Charry, Kevin M. Elias, Neil S. Horowitz, Michael Seckl, Ross S. Berkowitz

**Affiliations:** 1 Postgraduation Program in Tocogynecology of Botucatu Medical School, São Paulo State University Julio de Mesquita Filho - UNESP, Support Program for Foreign Doctoral Students (PAEDEx/UNESP) Botucatu, SP, Brazil; 2 Clinical Department, Universidad de Caldas, Manizales, Caldas, Colombia; 3 Botucatu Trophoblastic Disease Center of the Clinical Hospital of Botucatu Medical School, Department of Gynecology and Obstetrics, São Paulo State University Julio de Mesquita Filho - UNESP, Botucatu, SP, Brazil; 4 Carlos G Durand Hospital Trophoblastic Disease Center, Faculty of Medicine, Universidad de Buenos Aires, Buenos Aires, Argentina; 5 Oncólogos del Occidente S.A. Manizales, Caldas, Colombia; 6 Department of Obstetrics and Gynecology, Hospital Universitario de Caracas, Universidad Central de Venezuela, Caracas, Venezuela; 7 Division of Gynecologic Oncology, Department of Obstetrics, Gynecology and Reproductive Biology, New England Trophoblastic Disease Centre, Dana-Farber Cancer Institute, Brigham and Women's Hospital, Harvard Medical School, Boston, Massachusetts, United States; 8 Trophoblastic Tumour Screening and Treatment Centre, Charing Cross Hospital, Imperial College Healthcare NHS Trust, London, United Kingdom

**Keywords:** low-risk gestational trophoblastic neoplasia, molar pregnancy, South America, chemotherapy, resistance-related factors, neoplasia trofoblástica gestacional de baixo risco, gravidez molar, América do Sul, quimioterapia, fatores relacionados à resistência

## Abstract

**Objective** There are few multinational studies on gestational trophoblastic neoplasia (GTN) treatment outcomes in South America. The purpose of this study was to assess the clinical presentation, treatment outcomes, and factors associated with chemoresistance in low-risk postmolar GTN treated with first-line single-agent chemotherapy in three South American centers.

**Methods** Multicentric, historical cohort study including women with International Federation of Gynecology and Obstetrics (FIGO)-staged low-risk postmolar GTN attending centers in Argentina, Brazil, and Colombia between 1990 and 2014. Data were obtained on patient characteristics, disease presentation, and treatment response. Logistic regression was used to assess the relationship between clinical factors and resistance to first-line single-agent treatment. A multivariate analysis of the clinical factors significant in univariate analysis was performed.

**Results** A total of 163 women with low-risk GTN were included in the analysis. The overall rate of complete response to first-line chemotherapy was 80% (130/163). The rates of complete response to methotrexate or actinomycin-D as first-line treatment, and actinomycin-D as second-line treatment postmethotrexate failure were 79% (125/157), 83% (⅚), and 70% (23/33), respectively. Switching to second-line treatment due to chemoresistance occurred in 20.2% of cases (33/163). The multivariate analysis demonstrated that patients with a 5 to 6 FIGO risk score were 4.2-fold more likely to develop resistance to first-line single-agent treatment ( *p* = 0.019).

**Conclusion** 1) At presentation, most women showed clinical characteristics favorable to a good outcome, 2) the overall rate of sustained complete remission after first-line single-agent treatment was comparable to that observed in developed countries, 3) a FIGO risk score of 5 or 6 is associated with development of resistance to first-line single-agent chemotherapy.

## Resumo

**Objetivo** Existem poucos estudos multinacionais sobre os resultados do tratamento da neoplasia trofoblástica gestacional (NTG) na América do Sul. O objetivo deste estudo foi avaliar a apresentação clínica, os resultados do tratamento e os fatores associados a casos de quimiorresistência em NTG pós-molar de baixo risco tratados com quimioterapia de agente único de primeira linha em três centros sul-americanos.

**Métodos** Estudo multicêntrico de coorte histórica incluindo mulheres com NTG pós-molar de baixo risco com estadiamento International Federation of Gynecology and Obstetrics (FIGO) em centros de atendimento na Argentina, Brasil e Colômbia entre 1990 e 2014. Foram obtidos dados sobre as características do paciente, apresentação da doença e resposta ao tratamento. A regressão logística foi usada para avaliar a relação entre fatores clínicos e resistência ao tratamento de primeira linha com agente único. Foi realizada uma análise multivariada dos fatores clínicos significativos na análise univariada.

**Resultados** Cento e sessenta e três mulheres com NTG de baixo risco foram incluídas na análise. A taxa global de resposta completa à quimioterapia de primeira linha foi de 80% (130/163). As taxas de resposta completa ao metotrexato ou actinomicina-D como tratamento de primeira linha e actinomicina-D como tratamento de segunda linha após falha do metotrexato foram 79% (125/157), 83% (⅚) e 70% (23/33), respectivamente. A mudança para o tratamento de segunda linha por quimiorresistência ocorreu em 20,2% dos casos (33/163). A análise multivariada demonstrou que pacientes com pontuação de risco FIGO de 5 a 6 foram 4,2 vezes mais propensos a desenvolver resistência ao tratamento com agente único de primeira linha ( *p* = 0,019).

**Conclusão** 1) Na apresentação, a maioria das mulheres demonstrou características clínicas favoráveis a um bom resultado, 2) a taxa geral de remissão completa sustentada após o tratamento de primeira linha com agente único foi comparável à de países desenvolvidos, 3) um escore de risco FIGO de 5 ou 6 está associado ao desenvolvimento de resistência à quimioterapia de agente único de primeira linha.

## Introduction

Gestational trophoblastic neoplasia (GTN) is a malignant form of gestational trophoblastic disease with abnormal proliferation of placental trophoblastic cells that secrete persistent amounts of human chorionic gonadotropin (hCG). 1 The clinical presentation of GTN is variable depending on previous pregnancy type, disease extension and histopathological classification. Invasive mole and choriocarcinoma are very responsive to chemotherapy, and hCG values are usually well correlated with the volume of disease. On the other hand, placental site trophoblastic tumor and epithelioid trophoblastic tumor are relatively chemoresistant and produce less hCG compared with other forms of GTN. 2 In these cases, surgery is often the treatment of choice. 2

Gestational trophoblastic neoplasia can occur after any type of pregnancy. 3 However, the risk of developing GTN is highest in women with a molar pregnancy. In fact, between 50 and 80% of all GTN cases originate from a hydatidiform mole, and the remainder from a term/preterm pregnancy, miscarriage, or ectopic pregnancy. 4 Between 15 and 20% of patients with a complete hydatidiform mole (CHM) and 1 to 4% of those with a partial mole develop postmolar GTN. 3 These rates may differ from region to region, possibly reflecting differences in the hCG assays and criteria used for the diagnosis of GTN, 5 or even unavailability of demographic data on the entire population.

Gestational trophoblastic neoplasia cure rates are over 90% overall 6 and above 99% in some countries. 7 These good results can be explained by the adequate use of hCG as a biomarker, provision of patient care in specialized centers, 6 identification of prognostic factors for chemotherapy response, 8 and availability of active second- and third-line chemotherapy regimens. In 2000, the International Federation of Gynecology and Obstetrics and the International Society of Gynecological Cancer (FIGO Oncology Committee, 2002) 8 established a combined anatomic staging (stages I, II, III, and IV) and modified World Health Organization (WHO) risk-factor scoring system for classifying GTN as low (< 7) or high (≥ 7) risk for the development of resistance to single-agent chemotherapy.

First-line chemotherapy with either methotrexate (MTX) or actinomycin D (ActD) is the treatment of choice worldwide for patients with low-risk postmolar GTN (score < 7). 7 The most commonly used chemotherapy regimens are MTX (intravenous (IV) or IM), maximum of 25 mg/m ^2^ daily for 5 days repeated every 2 weeks 9 ; 50 mg MTX for 8 days (IM) on days 1, 3, 5, and 7, and with folinic acid (FA) rescue (0.1 mg/kg) on days 2, 4, 6, and 8 10 ; ActD 1.25 mg/m ^2^ (maximum of 2 mg) single IV dose every 2 weeks 11 ; and ActD 10 to 12 µg/kg IV for 5 days every 2 weeks. 12 It is important to emphasize that prompt chemotherapy treatment with appropriate regimens and fixed and timely intervals between chemotherapy cycles limits the development of resistance and the exposure of most patients to multi-agent chemotherapy. 13 14

There are few studies on GTN treatment response in South American patients, 15 16 17 18 and most of them are single-center, hospital-based. 19 The difficulties in carrying out multicentric studies in South American countries include socioeconomic and cultural diversities, as well as language barriers (Portuguese, Spanish, English). Given these disparities, the assessment of whether the results of first-line treatment with a single agent are similar or different from those observed in specialized centers in developed countries is relevant. Therefore, the purpose of this study was to assess the clinical presentation, treatment outcomes, and factors associated with chemoresistance in South American patients with low-risk postmolar GTN treated with first-line single-agent chemotherapy.

## Methods

This historical, multicentric, cohort study included women with FIGO-defined low-risk postmolar GTN 8 treated from 1990 to 2014 at one of the following South American centers: Centro de Doenças Trofoblásticas de Botucatu, Universidade Estadual de São Paulo —Unesp, São Paulo, Brazil; Durand Trophoblastic Diseases Center in Buenos Aires, Hospital Carlos G. Durand, Buenos Aires, Argentina; and Oncólogos del Occidente S.A. Manizales, Caldas, Colombia). These three centers provide tertiary care and consultation free of charge.

Patients with insufficient clinical information, lost to follow-up before 12 months after serum hCG normalization, having undergone primary hysterectomy, or initially treated outside the centers participating in this study were excluded.

In all study participants, metastases were detected by clinical, gynecological, and imaging examinations. First, transvaginal power Doppler sonography was performed to rule out the presence of pregnancy and residual molar tissue, and to assess the myometrium for invasion and pelvic mass vascularity. A chest X-ray including at least two views (posterior-anterior and lateral) was used to count lung metastases and FIGO scoring. When chest X-rays were normal, no further imaging was ordered, and treatment was promptly indicated. For patients with genital metastases, suspected lung metastasis on chest X-ray, or nodules > 1 cm, a chest tomography was conducted to determine the extent of disease in the lungs. 7 At each of the three participating centers, images of lung metastases were reviewed, and metastatic nodules ≥ 1 cm were counted. 7 Metastases at other sites were investigated as described elsewhere. 17

The hCG tests used for monitoring response to treatment were Abbott Architect (Centro de Doenças Trofoblásticas de Botucatu) and Roche Elecsys (Durand Trophoblastic Disease Center). At Oncólogos del Occidente S.A., kits by different manufacturers were used (Abbott Architect, Roche Elecsys, Siemens Immulite). However, all hCG tests for each individual patient were performed at the same laboratory to avoid variation among methods and results.

Participants received first-line chemotherapy with 8-day MTX/FA with FA rescue (0.1 mg/kg or 15 mg fixed dose, 10 13 5-day MTX, 9 5-day ActD or pulsed ActD each as a 2-week cycle. Multiple cycles of the same chemotherapy regimen were given until hCG remission, resistance development, or substantial toxicity (grades 3–4) (CTCAE, version 5) was reached. Consolidation chemotherapy was administered using the last effective regimen as one cycle to all patients in Argentina, while in Brazil and Colombia, two consolidations cycles were given for stage II or III cases. Patients were advised to use oral contraception throughout GTN treatment and follow up. 19 20

Remission was defined as 3 normal consecutive serum hCG measurements (< 5 IU/L for Brazil and Colombia; < 2 IU/L for Argentina) taken every 1 to 2 weeks. Cure or sustained complete remission was declared when normal values of hCG were observed for at least 12 months after remission was achieved. Resistance was determined by a hCG plateau of ± 10% over 2 weeks or re-elevation in at least one measurement of the hCG level while the patient was on first-line treatment with MTX or ActD. Second-line treatment consisted of switching the first single agent used (MTX as first-line switched for ActD as second line and vice versa) or capecitabine. 15 All patients either resistant to MTX or with MTX toxicity were eligible for ActD or capecitabine second-line therapy. Two patients received etoposide due to ActD unavailability, and three were given multiagent chemotherapy due to a higher FIGO score (score of 6) or hCG > 30,000 IU/L at the point of resistance to first-line single agent therapy.

The medical records (paper and electronic) of all patients with low-risk postmolar GTN (stages I–III, score < 7) were reviewed. Data on patient characteristics, disease presentation, and treatment response were obtained.

Patient and disease characteristics were assessed based on age, gravidity, parity, molar histology (complete or partial), time between molar evacuation and postmolar GTN diagnosis, interval between GTN diagnosis and chemotherapy initiation, hCG before first-line chemotherapy, presence of metastatic disease, and FIGO stage/risk score.

Response to first-line treatment with MTX or ActD was investigated using the following variables: number of first-line chemotherapy cycles, total number of chemotherapy cycles required to achieve remission, time to remission (interval between first-line chemotherapy initiation and first normal hCG), need for switching to second-line chemotherapy due to resistance to first-line treatment, or occurrence of substantial toxicity, surgery after chemoresistance, and survival rate.

This study was approved by the Research Ethics Committees of all participating institutions.

Statistical analyses were performed using the IBM SPSS Statistics for Windows, version 21.0 software (IBM Corp., Armonk, NY, USA). Univariate analysis was used to assess the associations between clinical factors and resistance to single agent first-line treatment. A multiple logistic regression model was fit to evaluate the associations of resistance to single agent first-line treatment with the clinical factors considered significant ( *p* < 0.20) on univariate analysis. Associations were considered significant when *p* < 0.05.

This study was approved by ther ethics committee of the Botucatu Medical School of Universidade Estadual de São Paulo, under protocol no. 563.812 (CAAE 17817613.6.0000.5411), Comité de Bioética Hospital General de Agudos Carlos G. Durand (DI-2018–534-HGACD), and Comité de Ética en Investigación de Oncolólogos del Occidente (IC-FO-003).

## Results

**Fig. 1:**
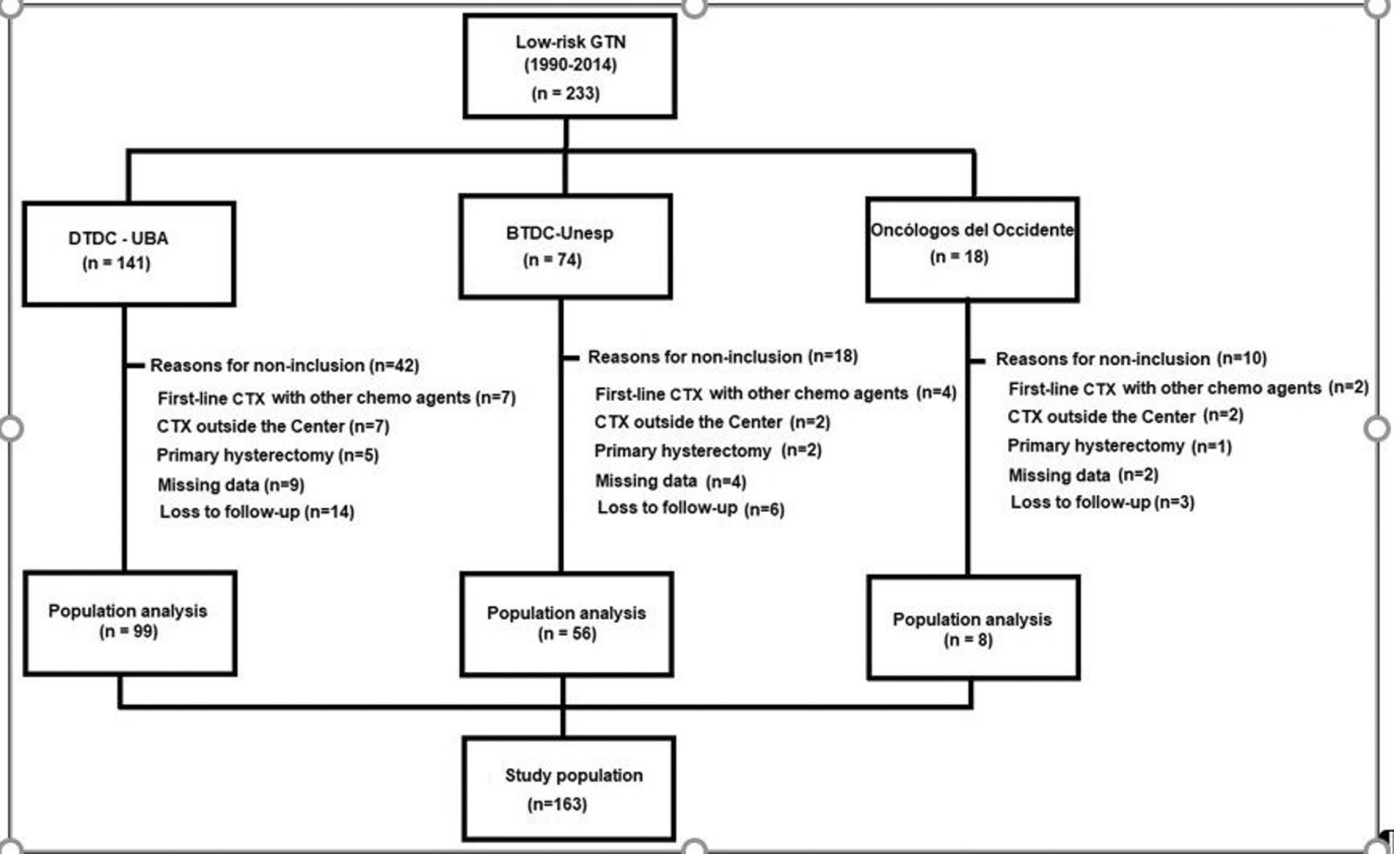
Patient flow chart.

Of 233 women diagnosed with FIGO-defined low-risk postmolar GTN, 163 received single agent first-line treatment with either MTX or ActD and were included in the analysis. The remaining 70 women were excluded for the following reasons: first-line treatment with chemotherapy agents other than MTX and ACTD ( *n* = 13), initial treatment outside the study Center ( *n* = 11), primary hysterectomy ( *n* = 8), missing data ( *n* = 15), and loss to follow-up < 12 months after serum hCG normalization ( *n* = 23) ( Fig. 1 ).

The clinical characteristics of the 163 study participants with low-risk postmolar GTN are shown in Table 1 . The median age (1st, 3rd quartile) of patients was 28 years (21, 33 years) and the median parity (1st, 3rd quartile) was 1 (0, 1). Complete hydatidiform mole was the most frequently observed type of mole (83.4%). The median interval between molar evacuation and postmolar GTN diagnosis (1st, 3rd quartile) was 2.3 months (1.6, 3.6), and hCG level at GTN diagnosis was < 100,000 IU/L in 94% of patients. At presentation, metastatic disease was observed in 51 patients (31.2%) (48 lung, 2 vagina, and 1 pelvis). Nearly 90% of the women had FIGO risk score < 5.

**Table 1:** Patient and disease characteristics ( *n* = 163)

Variables	Summary
Age (years)	28.0 (21.0, 33.0)
Gravidity	2.0 (1.0, 3.0)
Parity	1.0 (0.0, 1.0)
Molar histology	
Partial	27 (16.6%)
Complete	136 (83.4%)
HM evacuation-GTN diagnosis interval (months)	2.3 (1.6, 3.6)
GTN diagnosis- chemotherapy initiation interval (days)	2.0 (0.0–6.0)
Pre-treatment hCG	7,442 (1,693, 29,227)
Pre-treatment hCG	
< 1,000	31 (19.0%)
1,000 < 10,000	60 (36.8%)
10,000–99,999	62 (38.0%)
≥ 100,000	10 (6.2%)
Metastatic disease at presentation	51 (31.2%)
FIGO stage	
1	112 (68.7%)
2	3 (1.8%)
3	48 (29.4%)
FIGO risk score	3.0 (1.0, 4.0)
FIGO risk score	
0–2	81 (49.7%)
3–4	61 (37.5%)
5–6	21 (12.8%)
Number of first-line chemotherapy cycles	4.0 (3.0, 7.0)
Total number of chemotherapy cycles to remission	5.0 (3.0–8.0)
Time to hCG remission (weeks)	12.5 (7.7, 19.0)
Switch to second-line treatment	33 (20.2%)
Switch to third-line treatment	2 (1.2%)
Surgery after chemoresistance	11 (6.74%)
Survival rate	163 (100.0%)

Abbreviations: FIGO, International Federation of Gynecology and Obstetrics; GTN, gestational trophoblastic neoplasia; hCG, human chorionic gonadotropin; M, hydatidiform mole.

Data are median (25th–75th percentile) or n (%).

The following first-line single agent chemotherapy regimens were administered: 8-day MTX/FA ( *n* = 142), 5-day MTX ( *n* = 15), pulsed ActD ( *n* = 1), or 5-day ActD ( *n* = 5). Of note, the median interval between GTN diagnosis and initiation of single-agent first-line treatment (1st, 3rd quartile) was 2 days (0–6). The median number of cycles (1st, 3rd quartile) given until hCG normalization or first-line agent resistance was 4 (3, 7). The rates of sustained complete remission with MTX or ActD as first-line treatment, were 79.6%% (125/157) and 83.3% (⅚), respectively. Among the 33 patients (20.2%) requiring a switch to second-line treatment, 24 received ActD, 4 capecitabine, 2 etoposide, and 3 multiagent chemotherapy. Failure of second-line single-agent treatment was observed in only 2 patients (1.2%), 1 with 5-day ActD, and the other with capecitabine, who required multiagent chemotherapy to achieve complete remission. Surgery after chemoresistance was performed in 6.7% of patients (9 underwent hysterectomy, and 2 local uterine resection). The median time (1st, 3rd quartile) to reach hCG normalization was 12 weeks (7.7, 19). Gestational trophoblastic neoplasia patients with FIGO risk score 5 to 6 that achieved remission after first- or second-line single-agent chemotherapy accounted for 90.5% (19/21) of the study population ( Table 1 ).

Two patients experienced substantial toxicity during first-line treatment (grade 3 oral mucositis in 1 patient who received 5-day-MTX, and grade 3 nausea/vomiting in 1 patient who received 5-day ActD). However, no failure in first-line single-agent treatment due to chemotherapy-induced toxicity was observed. In these cases, chemotherapy was discontinued, and, because hCG normalization was reached while the patients were recovering from toxicity, chemotherapy was no longer used. All study participants were followed up for at least 12 months after completing chemotherapy. Survival rate between low-risk GTN diagnosis and the end of follow-up was 100% ( Table 1 ).

Univariate analysis showed that a FIGO risk score of 5 to 6 (odds ratio [OR] = 5.75 [95% CI 2.03–16.31], *p* = 0.001), total number of chemotherapy cycles required to achieve remission (1.32 [1.16–1.49], *p* < 0.001), and time to remission (1.07 [1.03–1.11], *p* = 0.001) were identified as significant indicators of resistance to first-line single-agent treatment ( Table 2 ). Multivariate analysis, including the clinical factors considered significant on univariate analysis, demonstrated that patients with a FIGO risk score of 5 to 6 were 4.2-fold more likely to develop resistance to first-line single-agent treatment ( *p* = 0.019). Among the patients with a 5 to 6 FIGO score, the rate of chemoresistance to first-line treatment was 52.4% (11/21), whereas in those with a FIGO risk score of 0 to 2 and 3 to 4, it was 16% (13/81) and 14.8% (9/61), respectively.

**Table 2:** Univariate associations of resistance to first-line chemotherapy with clinical factors

Variables	OR	95% CI		*P*
Age (years)	0.98	0.94	1.03	*0.488*
Gravidity	0.79	0.58	1.08	*0.146*
Parity	0.80	0.55	1.18	*0.260*
Complete mole	0.87	0.32	2.36	*0.780*
HM evacuation - GTN diagnosis interval (months)	0.97	0.82	1.16	*0.740*
GTN diagnosis-chemotherapy initiation interval (days)	0.97	0.92	1.03	*0.327*
Pretreatment hCG	1.00	1.00	1.00	*0.020*
Pretreatment hCG (Reference ≤ 1,000)				*0.140*
[1,000–10,000)	0.45	0.14	1.43	*0.178*
[10,000–100,000)	1.09	0.39	3.04	*0.863*
≥ 100,000	2.29	0.50	10.45	*0.286*
Metastatic disease at presentation	1.26	0.55	2.85	*0.584*
FIGO stage (reference = 1)				*0.715*
2	2.17	0.19	25.03	*0.536*
3	1.29	0.57	2.94	*0.547*
FIGO risk score	1.43	1.10	1.86	*0.007*
FIGO risk score (reference = 0–2)				
3–4	0.91	0.36	2.28	0.833
5–6	5.75	2.03	16.31	0.001
Number of first-line chemotherapy cycles	1.05	0.94	1.17	*0.405*
Total number of chemotherapy cycles to remission	1.32	1.16	1.49	*< 0.001*
Time to hCG remission (weeks)	1.07	1.03	1.11	*0.001*

Abbreviations: CI, confidence interval; FIGO, International Federation of Gynecology and Obstetrics; GTN, gestational trophoblastic neoplasia; hCG, human chorionic gonadotropin; HM, hydatidiform mole; OR, odds ratio.

## Discussion

This study including South American women with low-risk postmolar GTN showed that most patients had clinical characteristics favorable to a successful response to treatment. The overall rate of sustained complete remission after first-line single-agent treatment was ∼ 80%, and high FIGO scores (5–6) were strongly associated with resistance to first-line single-agent therapy.

The rates of sustained complete remission with MTX or ActD as first-line treatment were similar to those reported in developed countries 9 13 14 21 and other specialized South American centers as well. 16 18 The excellent overall cure rate found in our study is likely to be explained by the fact that our patients were initially treated in specialized centers. These provide interdisciplinary care, give each patient and their families the information needed to understand the disease and foster awareness of the importance of regular hCG monitoring, as well as actively identifying and recalling patients who miss appointments. 22 These centers perform GTN staging within 1 day after diagnosis so that prompt chemotherapeutic treatment can be started as recommended. 23 Additionally, these centers provide uninsured patients with medical care and chemotherapy drugs free of charge. 17 However, it is worth noting that specialized centers are not found in all Latin American countries, where the time elapsed between GTN diagnosis and treatment initiation is influenced by socioeconomic factors and health policies that do not always prioritize treatment for a rare condition such as GTN. 19 Centralized hCG monitoring has been recommended after molar evacuation, but this is not yet feasible in all countries and regions of South America. Therefore, training physicians for patient referral to a specialized center as soon as hCG plateaus or rises is essential to allow the prompt start of chemotherapy to patients with lower FIGO risk scores (< 5) to quickly achieve therapeutic success. 5 19

In the majority of our patients, disease duration was less than 4 months, serum hCG level was within the lower range, FIGO risk score was < 5, and the interval between GTN diagnosis and initiation of first-line single agent treatment was short, allowing an excellent response to treatment. Indeed, previous reports have demonstrated that, in women with GTN followed up at specialized centers after molar evacuation, median FIGO risk score is lower and median time interval between molar evacuation and chemotherapy initiation is shorter than in those initially treated in other institutions. 24

In our patients, treated with either MTX or ActD, the rate of switching to second-line therapy due to the development of resistance to first-line treatment was comparable to those reported with the use of MTX 9 21 and ActD as first-line therapy. 25

The single-agent ActD regimen has been reported to provide a higher complete remission rate than MTX. 26 However, MTX has been demonstrated to have a more favorable toxicity profile, 18 as it causes no alopecia and has no vesicant properties and reduced risk of vascular disorders, which have all been associated with ActD, particularly the 5-day regimen. 27 These data, added to the fact that ActD is not always available, have made multi-course MTX and rescue with folinic acid the most commonly used regimen in Latin America, where it is administered in an outpatient setting. 16 18 19 28

Multivariate analysis revealed that our patients with FIGO risk score of 5 to 6 are significantly more likely to develop resistance to first-line single-agent treatment. This is in line with other reports that show that around 70% of patients in this subset become chemoresistant, and that this is directly related to chemotherapy duration and need for switching to a subsequent more toxic treatment. 1 13 Notably, all our patients who experienced treatment failure with first-line single-agent chemotherapy were cured with sequential single-agent chemotherapy or multiagent chemotherapy with or without the use of salvage surgery.

Several previous investigations have identified pretreatment hCG > 100,000 IU/L, 29 uterine artery pulsatility index < 1, 30 and presence of lung metastases 31 as predictive of resistance to first-line therapy in women with low-risk GTN. However, none of these resistance predictors have been validated. 5 The largest international collaborative study of GTN patients with a FIGO score of 5 to 6 to date 4 reveals that metastatic disease, choriocarcinoma, and pretreatment hCG concentration are significant predictors of resistance to single-agent therapies among these women. 4 However, in our study, FIGO score 5 to 6 was the only factor to reach statistical significance. The fact that hCG at diagnosis was < 100,000 IU/L in the great majority of our patients might explain this finding.

Despite being multicentric, this study is limited by the inherent bias introduced by its retrospective hospital-based design which affect the generalizability of our results. It is noteworthy that the participating centers used different hCG cutoffs to define remission, but this did not affect the assessment of chemoresistance incidence. Other limitations included the small sample size (especially in the scenarios of risk factors for chemoresistance in case of low-risk GTN FIGO risk score of 5–6), and the fact that different first- and second-line regimens were used at the study centers. Additionally, although present in some studies used for comparison, factors related to chemoresistance in low-risk GTN, such as presence of choriocarcinoma, antecedent miscarriage, preterm/term gestation, and ectopic pregnancy, were not addressed herein. Nonetheless, the power of our multivariate analysis on the occurrence of resistance to first-line single-agent treatment was over 95%.

This is the first study to describe low-risk GTN clinical presentation, remission rates, treatment outcomes and resistance risk in South American gestational trophoblastic centers. Our findings may be useful to physicians in guiding decision-making, as they indicate which patients are at a higher risk of resistance and should, therefore, be referred to a specialized center upfront. This can contribute to improve the quality of life of the patients by decreasing exposure to more toxic multiagent therapy and increasing their chance to achieve complete remission more quickly.

## Conclusion

In brief, this study of South American women with low-risk of GTN treated in specialized centers led to the following conclusions: 1) at presentation, most patients showed clinical characteristics favorable for a good outcome, 2) the overall rate of sustained complete remission observed after first-line single-agent treatment was comparable to that of developed countries, 3) FIGO risk scores of 5 or 6 are associated with the development of resistance to first-line single-agent chemotherapy.
